# Trachelectomy and Cerclage Placement as Fertility-Sparing Surgery for Cervical Cancer—An Expert Survey

**DOI:** 10.3390/jpm15030077

**Published:** 2025-02-20

**Authors:** Anke Smits, Janneke T. Wolswinkel, Mieke L. G. ten Eikelder, Nadeem R. Abu-Rustum, Glauco Baiocchi, Jogchum J. Beltman, Allan Covens, Karlijn M. C. Cornel, Henrik Falconer, Christina Fotopoulou, Cornelis G. Gerestein, Blanca Gil-Ibanez, Peter Hillemanns, Christhardt Köhler, Ali Kucukmetin, Luc R. C. W. van Lonkhuijzen, Philippe Morice, Joo Hyun Nam, Myriam B. Perrotta, Jan Persson, Marie Plante, Denis Querleu, Reitan Ribeiro, Laszlo Ungár, Maaike A. P. C. van Ham, Petra L. M. Zusterzeel

**Affiliations:** 1 Department of Obstetrics and Gynecology, Radboud University Medical Center, 6500 HB Nijmegen, The Netherlands; 2 Gynecology Service, Department of Surgery, Memorial Sloan Kettering Cancer Center, New York, NY 10065, USA; 3 Gynecological Oncology, A.C. Camargo Cancer Center, São Paulo 01509-001, Brazil; 4 Department Obstetrics and Gynaecology, Leiden University Medical Center, 2300 RC Leiden, The Netherlands; 5 Odette Cancer Centre, Division of Gynecologic Oncology, University of Toronto, Toronto, ON M4N 3M5, Canada; 6 Department of Women’s and Children’s Health, Karolinska Institutet, SE-171 77 Stockholm, Sweden; 7 Department of Pelvic Cancer, Theme Cancer, Karolinska University Hospital, SE-171 76 Stockholm, Sweden; 8 Gynecological Oncology, Department of Surgery and Cancer, Imperial College London, London SW7 2AZ, UK; 9 Department of Gynecologic Oncology, University Medical Center Utrecht, Utrecht University, 3508 GA Utrecht, The Netherlands; c.g.gerestein-2@umcutrecht.nl; 10 Gynecologic Oncology and Minimally Invasive Surgery Unit, Gynecology and Obstetrics Department, University Hospital 12 de Octubre, 28041 Madrid, Spain; 11 Department of Gynecology and Obstetrics, Hannover Medical School (MHH), Carl-Neuberg-Str. 1, 30625 Hannover, Germany; 12 Department of Gynecology Asklepios Clinic Hamburg Altona, Paul-Ehrlich-Strasse 1, 22763 Hamburg, Germany; 13 DRK Clinic Berlin Westend, Spandauer Damm 130, 14050 Berlin, Germany; 14 Northern Gynecological Oncology Centre, Queen Elizabeth Hospital, Gateshead NE9 6SX, UK; 15 Center for Gynecologic Oncology Amsterdam, Department of Gynecology, Cancer Center Amsterdam, Amsterdam UMC, 1081 HV Amsterdam, The Netherlands; 16 Department of Gynecological Surgery, Inserm Unit 10-30, Gustave Roussy, 94805 Villejuif, France; 17 Department of Gynecologic Surgery, Gustave Roussy Cancer Campus, University Paris-Sud (Paris XI), 91405 Le Kremlin-Bicêtre, France; 18 Asan Medical Center, Department of Obstetrics and Gynecology, University of Ulsan College of Medicine, Seoul 44610, Republic of Korea; 19 Servicio de Ginecología, Hospital Italiano de Buenos Aires, Buenos Aires C1199ABB, Argentina; 20 Department of Obstetrics and Gynecology, Skåne University Hospital, SE-221 85 Lund, Sweden; 21 Department of Clinical Sciences, Faculty of Medicine, Lund University, SE-221 85 Lund, Sweden; 22 Department of Gynecological Oncology, Laval University, Quebec City, QC G1V 0A6, Canada; 23 UOC Ginecologia Oncologica, Dipartimento di Scienze della Salute della Donna, del Bambino e di Sanità Pubblica, Fondazione Policlinico Universitario Agostino Gemelli IRCCS, 00168 Rome, Italy; 24 Department of Gynecological Oncology, Erasto Gaertner Hospital, Curitiba 81520-060, Brazil; 25 Duna Medical Center, Department of Gynecology, 1095 Budapest, Hungary

**Keywords:** cervical cancer, fertility sparing surgery, cerclage, practice variation, minimal invasive surgery, trachelectomy

## Abstract

**Background/Objectives**: Fertility-sparing surgery (FSS) is a standard practice for managing early stage cervical cancer, yet significant variation exists in clinical approaches worldwide. Our objective was to ascertain current practices and preferences for cerclage use among expert centers globally regarding FSS in patients with early stage cervical cancer. **Methods**: We conducted a cross-sectional survey from May to July 2023 involving expert centers identified through their scientific contributions and participation in international workgroups and conferences.. The survey, comprising 27 questions, evaluated existing practices in FSS. **Results**: Out of the centers surveyed, 21 (36.2%) gynecologic oncologists responded. For tumors <2 cm, 86% of centers preferred radical trachelectomy, primarily via the vaginal approach, while 13.6% favored a simple trachelectomy. Three experts preferred simple trachelectomy (13.6%). For tumors >2 cm, 47.6% utilized neoadjuvant chemotherapy before trachelectomy. Others did not offer FSS or performed an abdominal radical trachelectomy. Over time, there has been a shift towards less radical surgeries for tumors <2 cm and increased use of neoadjuvant chemotherapy for larger tumors. Some abandoned the minimally invasive surgical approach. Nearly all experts (90.5%) placed a cerclage immediately following trachelectomy. **Conclusions**: The majority of experts opt for radical trachelectomy in early stage cervical cancer, with immediate cerclage placement being a common practice. However, considerable international variations highlight the urgent need for standardized guidelines and further research to optimize treatment strategies, balancing oncological safety with fertility outcomes.

## 1. Introduction

Nearly half of women diagnosed with cervical cancer are younger than 45 years, with a significant proportion wishing to preserve their fertility [1]. The mean age for a woman’s first childbirth is increasing in many countries, currently 30 years in the United States [2]. This results in an increasing number of women with cervical cancer before having their first child. Currently, for tumors less than 2 cm (FIGO 2018 stage IA1, stage IA2, and IB1), fertility-sparing surgery (FSS) is considered safe and offered as part of standard practice [3,4]. For stage IA1 disease, surgery is usually limited to a loop excision or conization, with possible assessment of pelvic node status depending on the presence of lymphovascular space invasion (LVSI). For stage IA2 and IB1 disease, fertility-sparing treatment usually comprises a simple or radical trachelectomy, depending on tumor size, histology, LVSI, and resection margins of conization, combined with pelvic node staging [5]. Women with FIGO stage IB2 disease (tumor size 2–4 cm) may also be considered for FSS, although evidence is limited on oncological safety for FSS in tumors >2 cm [6,7].

Pregnancy rates after FSS are significantly lower compared to the general population, with reported rates between 36 and 66%, and these pregnancies are at increased risk of preterm birth due to premature prelabor rupture of membranes (PPROM), infection, and/or cervical insufficiency [6,8]. In an attempt to decrease these risks, it is important to preserve as much cervical tissue without compromising oncological safety, with a consequent shift towards less radical surgery [9]. Following the reported higher pregnancy rates after radical vaginal trachelectomy, several centers have shifted towards a vaginal approach instead of performing an open abdominal radical trachelectomy [6]. The paucity of comparative research in FSS has led to the extrapolation of outcomes of other cervical cancer trials to guide clinical practice [5]. The recent SHAPE trial showed that a simple hysterectomy instead of a radical hysterectomy for tumors less than 2 cm did not compromise oncological outcomes [10]. These results may potentially be extrapolated to the context of FSS since the risk for parametrial involvement is low in small tumors [11]. Minimally invasive surgery (MIS) for FSS has been abandoned by some centers following outcomes of the LACC trial, despite the fact that FSS patients were not included in the trial [12]. Prospective evidence on oncology safety of MIS in FSS is lacking; a systematic review of retrospective studies, including the IRTA study, showed no difference in survival rates between open abdominal radical and minimally invasive radical trachelectomy [13,14].

Current guidelines suggest the placement of a permanent cerclage around the remaining cervix after trachelectomy irrespective of approach, with the aim of reinforcing the cervix in the event of a future pregnancy and minimizing the risk for miscarriage or preterm birth [5]. However, there is insufficient evidence showing that cerclage placement prevents PPROM, infection, and cervical insufficiency after FSS. However, since cerclage placement can lead to cervical stenosis and erosion, some centers have abandoned routine cerclage placement after trachelectomy [15,16].

The lack of comparative studies or randomized controlled trials has resulted in significant variations between centers in the practice of both FSS and cerclage placement after surgery. The relatively new FIGO staging, as well as studies showing that less radical surgery may be safe, may have even led to more diversity in clinical practice. To gain insight into current clinical practice, we performed an international survey among clinicians working in expert centers. The survey aimed to assess the current practice of trachelectomy and subsequent placement of cerclage for early stage cervical cancer.

## 2. Materials and Methods

### 2.1. Study Design

A cross-sectional survey study was performed among trachelectomy expert centers worldwide. The identification of eligible expert centers was carried out through a comprehensive process involving a systematic review of relevant literature. The search of the literature has been described elsewhere and included in Appendix A [11]. A total of 54 expert centers were selected which had described and/or performed a case series with similar approaches to fertility-sparing surgery through trachelectomy for cervical cancer. This study was exempted from ethical approval by the institutional review board, in concurrence with the Medical Research Involving Human Subjects Act (2022-16005).

### 2.2. Procedure

Affiliated gynecological oncologists, key opinion leaders from these expert centers, were approached by email between May 2023 and July 2023 and sent a personalized link to the survey. The survey was administered in English and collected through Castor, an electronic database. Those who did not complete the survey were sent a maximum of three additional invitations to participate. Participation was voluntary, without any incentive or compensation offered.

### 2.3. Survey

The survey comprised questions assessing demographic characteristics and questions assessing current and previous practices of trachelectomy and cerclage placement. The original questionnaire is detailed in Appendix B. Respondent baseline characteristics such as age, gender, center of practice, years of experience, and number of annual procedures performed were collected through the survey. Current practices were assessed using multiple choice questions with the possibility to expand or comment on answers given. Survey questions were reviewed by four clinicians (AS, MtE, MvH, and PZ) prior to their administration. Questions varied from binary questions (Yes/No) to multiple choice questions and open questions. Experiences and views of experts regarding several aspects of trachelectomy and cerclage placement were also assessed through statements which could be answered with “yes”, “no”, or “I don’t know”. Respondents were given the opportunity to comment on the survey and recommend other experts for participation in this survey. Following feedback from respondents, an additional three questions were added to the survey, for which an additional invitation was sent to all initial respondents (Appendix B).

### 2.4. Data Analyses

Data were analyzed using SPSS version 27. Descriptive statistics were calculated using the number of responses. Continuous variables were presented as means with standard deviations or medians and interquartile ranges, as appropriate. Categorical outcomes were presented as frequencies and proportions.

## 3. Results

Questionnaires were sent out to 58 key opinion leaders in gynecological oncology from 54 expert centers. In total, 21 senior gynecological oncologists from 21 different centers for gynecological oncology completed the survey, resulting in a response rate of 36.2%. Demographic and baseline characteristics are detailed in Table 1. Almost all experts work in a tertiary referral center, and the majority are 50 years old or older (n = 13, 61.9%), with more than 10 years of experience.

### 3.1. Trachelectomy

All experts had more than 10 years of experience in performing trachelectomies. More than half of the experts performed less than 10 trachelectomies per year (n = 13; 61.9%), five experts (23.8%) performed 10–20 trachelectomies, and three experts (14.3%) performed 20–30 trachelectomies annually.

Assessment of cervical length and eligibility for a trachelectomy was conducted using MRI scan in almost all expert centers (n = 19, 90.5%). Eight experts used MRI alone, nine experts combined MRI with transvaginal ultrasound (n = 5) or clinical examination (n = 4), and two experts used a combination of the three. One expert only used transvaginal ultrasound for cervical assessment, and one described a combined approach of ultrasound and hysteroscopy to assess endocervical infiltration and thereby to determine the resection line prior to surgery.

The minimal cervical length (prior to surgery) allowing a trachelectomy was reported to be 1 cm (n = 6, 28.6%) or 2 cm (n = 8, 38.1%). One expert preferred a minimum length of 3 cm. Five experts did not specify a cut-off but stated that it depended on the histology of the previous cone (n = 1), tumor size (n = 3), and tumor localization (n = 1). Most experts aimed to have a minimal remaining cervical length of 0.5 cm (n = 10, 47.6%) to 1 cm (n = 9, 42.9%) after trachelectomy.

#### 3.1.1. Tumors Up to 2 cm

Almost all experts (n = 18, 85.7%) still preferred a radical trachelectomy for (FIGO 2018 IA2 or IB1) tumors ≤2 cm. Vaginal radical trachelectomy was the preferred approach (n = 9, 40.9%), followed by abdominal approach (n = 6, 28.5%) and robot-assisted approach (n = 3, 14.3%). Three experts specified that they preferred a simple vaginal trachelectomy for these tumors. Five experts believed that surgical approach may impact survival, with two stating that after the LACC trial, the laparoscopic approach should be abandoned.

#### 3.1.2. Tumors of More Than 2 cm

There was considerable variation in treatment practice for tumors >2 cm. Four experts (19.0%) indicated they did not offer fertility-sparing surgery for tumors more than 2 cm. Of the experts that did perform FSS in cervical tumor >2 cm, nine experts reported the use of NACT followed by a cone or simple trachelectomy (n = 4, 19.0%), a radical vaginal trachelectomy (n = 3, 14.3%), or an abdominal radical trachelectomy (n = 2, 9.5%). In general, NACT was used when tumor size was more than 2 cm, but one expert specified a minimal diameter for NACT of 3 cm. Six experts did not give NACT but would offer an abdominal radical trachelectomy for tumors more than 2 cm. One expert also administered NACT followed by FSS for tumors more than 4 cm (FIGO stage IB3 disease). In addition, one expert specified that they only performed FSS for selected cases, but did not further specify their preferred type and approach.

#### 3.1.3. Evolution of Practice Over Time

When asked whether the preferred method or approach had changed over time, ten experts (47.6%) indicated that there had been a clear change in practice. Full explanations are detailed in Table 2. Two experts stated that they moved away from laparoscopic surgery towards abdominal trachelectomy since the LACC trial was published. A shift towards less radical surgery by performing a simple trachelectomy or even conization was frequently mentioned (n = 6, 28.6%) for low-risk tumors (1A2 or IB1) without LVSI as an alternative to a radical trachelectomy. Two experts moved from upfront radical trachelectomy towards administering NACT for larger tumors (stage IB2 disease), but one expert did exactly the opposite.

### 3.2. Cerclage Placement

In 19 centers, placing a cerclage after radical trachelectomy is considered standard practice. More than half of the experts (n = 13, 61.9%) believed that cervical insufficiency after trachelectomy is the main reason for preterm birth, while eight experts (38.1%) stated that infection was the main cause. Reasons for cerclage placement were to prevent preterm birth (n = 10, 47.6%) or midterm pregnancy loss (n = 6, 28.6%) or ‘better to be safe than sorry’ (n = 3, 14.3%). The most frequently used suture material was a monofilament (52.4%), and one specified using Gore-tex (4.5%). Arguments for suture choice are detailed in Table 3.

Seventeen experts encountered problems after placement of a cerclage. The most frequently mentioned cerclage complications by experts were erosion and/or expulsion (n = 10, 47.6%), persistent discharge (n = 5, 23.8%), infection (n = 2, 9.5%), dyspareunia (n = 1, 4.8%), cervical stricture/stenosis (9.5%), and tearing (4.8%). Five experts reported no problems encountered, all of them using a monofilament suture.

Two experts (9.5%) have abandoned cerclage placement, with a case load of 0–10 and 20–30 trachelectomies per year, as they believe there is no evidence for a benefit in pregnancy outcomes and cervical insufficiency is unlikely if there is sufficient remaining length of cervix after trachelectomy. Both experts have not noticed a change in incidence of midterm pregnancy loss nor preterm birth after this change in practice.

A total of 18 experts completed the additional three survey questions. All agreed that the optimal timing for cerclage placement was immediately following trachelectomy and most centers believed that route of placement did not affect fertility, obstetric outcomes, nor survival. In the case of a cerclage placement during pregnancy, ten (47.6%) preferred the abdominal route and eight (38.1%) the vaginal route.

## 4. Discussion

With this study, we have assessed the clinical practice of FSS expert centers worldwide and cerclage placement in early stage cervical cancer. FSS is an important treatment option for women with early stage cervical cancer who wish to preserve their fertility. Considerable variation in clinical practice exists, mostly because of the lack of comparative studies, leaving room for the surgeon’s experience as a treatment denominator.

Current guidelines mention vaginal, open abdominal, and minimally invasive approaches as options for performing a radical trachelectomy, without detailing further preference [5]. We noted significant variation among the surveyed experts, with the vaginal approach being the preferred method for tumors smaller than 2 cm. Hypothetically, the vaginal approach is thought to better preserve cervical functioning, as only the infra-ureteral parametrium is resected [17,18]. The abdominal approach usually consists of a larger resection of cervical stroma, the supra-ureteral lateral parametrium, and more pelvic splanchnic nerves, and sometimes include bilateral uterine arteries [17,18]. This might result in a reduced function of the uterus. Smith and all showed, in a review, higher pregnancy rates for the vaginal approach compared to the abdominal approach [19]. A recent updated review by Morice et al. comparing vaginal and abdominal open and minimal invasive approaches, with the majority being conventional laparoscopy, demonstrated comparable recurrence and survival between all approaches for tumors ≤2 cm. However, higher pregnancy rates were found after radical vaginal trachelectomy (59%) compared to open abdominal radical trachelectomy (36%) and minimal invasive trachelectomy (46%), with similar live birth and prematurity rates across the different approaches [6]. A recent large case series of 149 patients undergoing a robotic radical trachelectomy showed a pregnancy rate of 80% and a live birth rate of 70% [20]. Despite the lack of comparative trials, this data suggests a benefit for the vaginal and robot-assisted approach for tumors <2 cm.

In patients with tumors between 2 to 4 cm (stage IB2 disease), FSS is not a standard treatment but should be considered carefully according to ESGO guidelines [5]. In our survey, most experts preferred NACT prior to FSS for these tumors, while some performed an abdominal radical trachelectomy alone. Over the last years, NACT followed by a vaginal procedure (radical/simple trachelectomy or conization) has emerged as an alternative option to the abdominal radical trachelectomy due to its low pregnancy rates of 36% [6]. Van Kol et al. performed a systematic review on fertility and recurrence rates in patients with FIGO stage IB2 disease treated with an abdominal radical trachelectomy versus NACT followed by vaginal trachelectomy. They reported comparable oncological outcomes: after NACT, a recurrence rate of 10% and a death rate of 2.9%, and after abdominal trachelectomy, a recurrence rate of 6.9% and a death rate of 3.4%. However, they found significantly higher pregnancy rates in the patients treated with NACT followed by a vaginal trachelectomy compared to upfront abdominal trachelectomy of 70% compared to 21%, respectively [21]. Ongoing trials such as the CONTESSA trial will hopefully provide further insight for this specific group [22].

Given the fact that recurrence and survival data are sparse for FSS for tumors >4 cm, and derived from answers and free text quotes in our survey, we presume that most centers involved in our study do not performed FSS for these patients [23]. In our survey, we focused our questions on tumors smaller or larger than 2 cm. However, at least one expert in our survey mentioned performing radical trachelectomy after NACT for patients with tumors of 4 cm or larger (stage IB3).

An paramount part of patient selection for trachelectomy is based on the assessment of cervical length preoperatively, as it is important to maintain sufficient cervical length for child-bearing potential. However, there are no recommendations for the minimal cervical length that would be required for a trachelectomy. In our survey, required cervical length by experts varied mainly between 1 to 2 cm, although 24% of experts did not use a specific cut-off but based management on the tumor characteristics. Regarding the remaining cervical length after trachelectomy, in our survey, most experts aimed to have a minimal remaining length of 0.5 cm to 1 cm. For oncological safety, most studies suggest a minimum microscopic free margin of 5 mm, but consensus is lacking [24]. Intraoperative frozen section analysis of the upper resection margin needs to be considered to confirm adequate margins and ensure maximal cervical tissue preservation and consequently cervical functioning [5].

From obstetrical perspective, there are no recommendations regarding the minimal cervical length that should be preserved. As premature labor is observed in 18–30% of patients after conization or trachelectomy, it is assumed that the amount of cervix removed correlates with the increased risk of adverse obstetric outcomes [6]. Studies in patients with cervical intraepithelial neoplasia or early stage cervical cancer (IA1) showed a relationship between the volume excised from the cervix and the risk of preterm birth [25,26,27]. In addition, a study by Alvarez et al. demonstrated that a residual cervix length of <10 mm after radical vaginal trachelectomy was associated with a significantly increased risk for preterm birth (67%) and PPROM (37%) compared to patients with a cervix length of >10 mm (22% preterm birth, no risk of PPROM) [9]. Moreover, interestingly, Ekdahl et al. showed that the use of a second trimester oral metronidazole and no sexual intercourse regime may reduce second trimester miscarriage and premature deliveries after radial trachelectomy [28].

Removal of the parametria may cause denervation of the inferior hypogastric plexus, resulting in bladder and bowel complaints and sexual dysfunction. In addition, it may further negatively impact cervical functioning [29]. Consequently, the possibility and safety of omitting the parametrectomy for patients with small tumors has been debated for several years. Although there is a shift towards less radical surgery for patients with low-risk tumors smaller than 2 cm, our survey showed that most experts (86%) still preferred a radical trachelectomy. A recent review of 4118 women undergoing FSS for stage IA1, IA2, or IB1 disease (FIGO staging 2009) showed that the incidence of parametrial involvement was only 0.3% [11]. In addition, Zhang et al. performed a systematic review including patients with stage IA1-IB1 and found low recurrence rates of 0.4% in 347 patients treated with conization (n = 176 stage IA1, n = 30 stage IA2, and n = 167 stage IB1) [30]. A more recent review by Nezhat et al., including 3044 patients who underwent any type of FSS for stage IA1-IB1 disease, did not find significant differences in oncological outcomes between different approaches [31]. The results of the SHAPE trial confirmed, in a randomized controlled trial, the oncologic safety of a simple hysterectomy compared to a radical hysterectomy for cervical tumors <2 cm [10]. However, the trial did not distinguish between different types of histology nor the presence of lympho-vascular space invasion, limiting the applicability for certain subgroups. In accordance with the recently published observational study by Slama et al., we believe that many women with early stage cervical cancer can be treated less radically than has been done in the past [32]. We therefore propose a simple radical trachelectomy as management for low-risk IB1 tumors with limited stromal invasion (<10 mm; SHAPE trial inclusion criteria) while continuing the practice of a radical trachelectomy for the other 1B1 tumors [10].

Over the last decades, there has been a tremendous increase in the use of minimal invasive surgery, which can also be seen in cervical cancer surgery. However, with the LACC trial reporting decreased survival rates with MIS compared to open surgery for radical hysterectomy, this practice has widely been abandoned [12]. These outcomes have been attributed to several factors including type of MIS, as the trial mainly included conventional laparoscopy, tumor size, tumor manipulation, CO_2_ pneumoperitoneum, and the effect of surgical expertise and learning curves [12]. Future trials are already on their way, with modifications of the surgical technique and careful selection of patients with smaller tumors, prior conization, and minimal tumor manipulation using protective measures such as vaginal closure and avoiding uterine manipulators [33,34,35]. However, these concerns have been translated to FSS, even though this population was not included in this trial. This has resulted in a change of practice for trachelectomy, with several experts moving away from MIS and back to the open or vaginal approach [6]. However, MIS for trachelectomy has been developed as a surgical technique only recently [36]. The retrospective multi-institutional IRTA study by Salvo et al. is one of the few studies evaluating disease-free and overall survival after open versus minimally invasive trachelectomy in a multi-institutional setting. They showed that disease-free survival rates did not differ between open radical trachelectomy and minimally invasive radical trachelectomy. However, recurrence rates in each group were low [14]. In addition, a recent systematic review and meta-analysis including more than 1000 patients evaluated outcomes of MIS trachelectomy versus abdominal trachelectomy and found no significant differences in overall survival, tumor recurrence, or mortality rates. However, they did find lower pregnancy rates after a MIS trachelectomy compared to abdominal trachelectomy (31.3% versus 51.5%, respectively) [13]. However, the majority of MIS encompassed conventional laparoscopy, and therefore a further distinction needs to be made for robotic surgery, as the previously mentioned case series of Ekdahl et al. reported much higher pregnancy rates [20]. Ongoing prospective studies of conservative management of early stage cervical cancer may help guide future management. In addition, currently ongoing trials such as the RACC, ROCC, and LAUNCH 2 trials, comparing robot-assisted laparoscopic radical hysterectomy with laparotomic radical hysterectomy, may also provide further insight on the safety of robotic MIS, despite not including FSS [33,34,35].

The need for FSS is changing over time as the impact of evolving screening practices and HPV vaccination could reduce the incidence of cervical cancer and therefore the demand for FSS. However, since FSS is most applicable in settings where early stage cancer can be detected, it is mainly limited to countries with organized or high-coverage opportunistic screening programs.

### 4.1. Cerclage Placement

To overcome the potential complications of FSS, cervical cerclages have been widely used and are currently still recommended in ESGO guidelines [5]. A cerclage aims to prevent further reduction of cervical length by improving mechanical support and possibly reduce the loss of cervical mucus production [37,38]. It is hypothesized that cervical mucus plays an important role in the prevention of chorioamnionitis, PPROM, and spontaneous preterm birth, as most second trimester miscarriages and preterm birth seem to be related to infection [8,39]. However, the effectiveness and safety of cerclage placement remain controversial, and this is also reflected by this survey. No comparative studies are available supporting cerclage placement, and some even suggest that a cerclage might contribute to complications such as cervical stenosis, erosion, and excessive vaginal discharge [15,16,40,41]. Despite these controversies, our study demonstrates that the majority of expert centers still routinely place cerclages immediately after trachelectomy. Two experts stopped performing cerclage placement due to insufficient evidence supporting its use, with the conviction that in case of sufficient remaining cervical length, cervical incompetence is unlikely.

**Figure 1:**
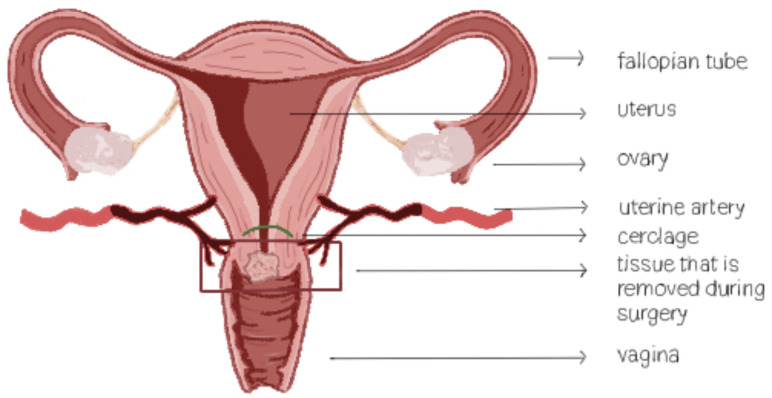
Location of cerclage placement.

In case of cerclage placement, a running suture is placed at the level of the isthmus (Figure 1). Both monofilament and multifilament suture material could be used for cerclage placement. Monofilament sutures give a low risk of infection. Multifilament sutures are strong and easy to place, but spaces between the suture strands could provide an reservoir for bacteria [42]. In the literature, we could not find an association between suture material and the occurrence of cervical stenosis or the incidence of pregnancy loss [43].

### 4.2. Strength and Limitations

This is the first study to provide an overview of the current practice of FSS and cerclage placement in expert centers worldwide. Although a systematic approach was used to identify expert centers through the literature and conferences, the risk of selection bias is still present. In addition, despite the fact that the response rate of 36% was comparable to that of other survey studies, this may represent further bias [44]. Lastly, our survey mainly comprised multiple choice questions, through which we run the risk of oversimplifying indications, practices, and outcomes. Respondents could only answer with our predefined answer options, which may have led to a loss in nuance that is applied in the real world.

## 5. Conclusions

Most expert centers continue to favor radical trachelectomy for managing early stage cervical tumors, with immediate cerclage placement remaining prevalent. Notably, there is a trend towards less radical surgical approaches over time, influenced by evolving insights into the balance between oncological safety and fertility preservation. These findings highlight the critical need for more standardized guidelines, which should be supported by data from prospective and randomized studies to define the optimal treatment strategies. Although initiating randomized trials in FSS presents substantial challenges, including difficulties in patient recruitment and the variability of outcome measures like disease recurrence and surgeons’ preferences, developing consensus guidelines appears to be the most practical approach to improve and standardize care. These guidelines should aim to refine FSS practices based on emerging evidence and expert consensus.

## Figures and Tables

**Table 1:** Characteristics of respondents.

Characteristics	Population = n 21 (%)
Country of practice	
Argentina Brazil Canada France Germany Hungary Italy Netherlands South Korea Spain Sweden United Kingdom USA	1 (4.8%) 2 (9.5%) 2 (9.5%) 1 (4.8%) 2 (9.5%) 1 (4.8%) 1 (4.8%) 4 (19%) 1 (4.8%) 1 (4.8%) 2 (9.5%) 2 (9.5%) 1 (4.8%)
Type of center	
Tertiary referral center Other: -Comprehensive cancer center -Private hospital with connection to a medical university center	19 (90.5%) 2 (9.5%)
Age 40–50 years 50–60 years 60 years or older	8 (38.1%) 4 (19.0%) 9 (42.9%)
Gender Male Female	16 (76.1%) 5 (23.8%)
Years in practice 5–10 years More than 10 years	2 (9.5%) 19 (90.5%)

**Table 2:** Expert responses to whether there was a change of practice over time.

1. I used to perform laparoscopic surgery before the LACC trial but now perform open surgery (n = 2). 2. I moved away from upfront radical trachelectomy in lesion ≤2 cm in favor of simple trachelectomy. In addition, I moved away from radical trachelectomy in lesions >2 cm in favor of NACT (n = 2). 3. I am reducing vaginal radicality towards simple trachelectomy. 4. I moved towards surgery without parametrial resection in low-risk tumors. 5. I moved from NACT to abdominal radical trachelectomy for stage IB2 disease, and moved from VRT to simple conization in stage IB1 without LVSI. 6. Change in approach to tumor size (not further specified). 7. FIGO 2009 IB1 > Figo 2018 IB1: radical vaginal trachelectomy, FIGO 2009 IB1 > FIGO 2018 IA1 or IA2: simple trachelectomy. 8. Simple trachelectomy more frequently used in tumors ≤2 cm, but size is not the only parameter.

**Table 3:** Reasons for selection of suture material for trachelectomy.

Multifilament (e.g., Mersilene, Tevdek, Ethibond) n = 9 (42.9%z%)	Monofilament (e.g., Prolene, Nylon) n = 11 (52.4%)	Other Gore-Tex 0 n = 1 (4.5%)
Specified reasons: - Ease of use - Strength - Reliability - Less likely to cut	Specified reasons: - Less associated with rejection (eruption/erosion) compared to multifilament - Less reaction of vaginal tissue - Soft to prevent vaginal irritation - Less traumatic - Lower risk of infection - Ties easily	Specified reasons: - Soft material - Small suture ties easily - Less likely to erode

## Data Availability

No new data were created or analyzed in this study.

## Appendix A

Search strategy
Part 1 (“Uterine Cervical Neoplasms”[Mesh] OR adeno squamous cell carcinoma*[Title/Abstract] OR cervical adenocarcinoma*[Title/Abstract] OR cervical cancer*[Title/Abstract] OR cervical carcinoma*[Title/Abstract] OR cervical neoplas*[Title/Abstract] OR cervical squamous cell carcinoma*[Title/Abstract] OR cervical tumo*[Title/Abstract] OR adeno squamous cell carcinoma*[Title/Abstract] OR cervix adenocarcinoma*[Title/Abstract] OR cervix cancer*[Title/Abstract] OR cervix carcinoma*[Title/Abstract] OR cervix neoplas*[Title/Abstract] OR cervix squamous cell carcinoma*[Title/Abstract] OR cervix tumo*[Title/Abstract]) AND Part 2 (“Conization”[Mesh] OR “Trachelectomy”[Mesh] OR “Organ Sparing Treatments”[Mesh] OR ((“surgery” [Subheading] OR “Gynecologic Surgical Procedures”[Mesh:NoExp]) AND (“Fertility Preservation”[Mesh] OR “Fertility”[Mesh] OR “Cervix Uteri”[Mesh])) OR cervicectom*[Title/Abstract] OR cone biops*[Title/Abstract] OR Conisation*[Title/Abstract] OR Conization*[Title/Abstract] OR cone resection [Title/Abstract] OR fertility preserv* [Title/Abstract] OR fertility sparing [Title/Abstract] OR Large loop excision of the transformation zone[Title/Abstract] OR LLETZ[Title/Abstract] OR trachelectom*[Title/Abstract]) AND Part 3 (“Lymph Nodes”[Mesh] OR “Lymph Node Excision”[Mesh] OR “Sentinel Lymph Node”[Mesh] OR lymph node* [Title/Abstract] OR Lymphadenectom*[Title/Abstract] OR sentinel node* [Title/Abstract] OR parametri*[Title/Abstract])

## Appendix B

Questionnaire
Q1. What is your country of practice?
Q2. What is the name of the hospital you work in?
Q3. In what type of center do you work?
  - Secondary referral center
  - Tertiary center
  - Other:
Q4. What is your age?
  - 30–40 years
  - 40–50 years
  - 50–60 years
  - 60 years or older
Q5. What is you gender?
  - Male
  - Female
  - Prefer not to say
  - Prefer to self-describe
Q6. How many years have you been in practice?
  - Less than 5 years
  - 5–10 years
  - More than 10 years
Q7. What is your preferred method for trachelectomy for tumors <2 cm?
  - Vaginal radical trachelectomy
  - Abdominal radical trachelectomy
  - Robot-assisted radical trachelectomy
  - Simple trachelectomy (without parametria)
Q8. What is your preferred method for trachelectomy for tumors of 2 cm or more?
  - Neoadjuvant chemotherapy followed by cone or simple trachelectomy
  - Neoadjuvant chemotherapy followed by radical vaginal trachelectomy
  - Abdominal radical trachelectomy
  - Robot-assisted radical trachelectomy
  - Other approach:
Q9. Has your preferred method/approach changed over time?
  - Yes Please explain how your practice has changed:
  - No
Q10. How many fertility preserving radical) trachelectomy procedures for cervical cancer (stages IA1/2 with LVSI to IB2) are performed annually at your health institution?
  - 0–10 cases
  - 10–20 cases
  - 20–30 cases
  - >30 cases
Q11. For how many years has your unit been performing radical trachelectomy procedures?
  - 0–5 years
  - 5–10 years
  - More than 10 years
Q12. How do you assess the cervical length and eligibility for a trachelectomy and cerclage placement? (Multiple answers possible)
  - Transvaginal ultrasound
  - MRI scan
  - Clinical examination (including examination under anesthesia).
  - CT scan
  - Other:
Q13. What do you consider is the minimal cervical length to be able to perform a trachelectomy?
  - Less than 1 cm
  - 1 cm
  - 2 cm
  - 3 cm
  - Other:
Q14. What do you consider the minimum remaining length of cervix for successful placement of a cerclage after radical trachelectomy?
  - 0.5 cm
  - 1 cm
  - 1.5 cm
  - Other:
Q15. Which statement best reflects the practice at your center?
  - Placing a cerclage after a radical trachelectomy is considered standard practice.
  - Placing a cerclage after a radical trachelectomy is occasionally performed and highly individualized care.
  - Cerclages are never placed after trachelectomy in our center.
  - In the past, we used to place cerclages after trachelectomy but not anymore.
If Q15. was answered with d.
Q15.1. What was the main reason for never performing or discontinuing placing cerclages after a radical trachelectomy?
  - There is no evidence for cervical insufficiency after a radical trachelectomy.
  - There is no evidence for a benefit in pregnancy outcomes.
  - The complications of the procedure outweigh its benefits.
  - Other reason being: ….
Q15.2. Has, to your knowledge, abandoning cerclages led to a change in incidence of midterm pregnancy loss or preterm birth?
  - Yes
  - No
  - I don’t know
Q16. What is/was the main reason for placing a cerclage at your center?
  - To prevent midterm pregnancy loss.
  - To prevent preterm birth.
  - Better to be safe than sorry.
  - Other reason:
Q17. What suture material is used for a cervical cerclage after the radical trachelectomy at your center?
  - Multifilament (e.g., Mersilene, Tevdek, Ethibond)
  - Monofilament (e.g., Prolene, Nylon)
  - Other, please specify
Q18. If possible, please specify the reason for choice of suture material.
Q19. What is your most frequently encountered problem/complication with cerclages? (Multiple answers possible).
  - Eruption
  - Infection
  - Persistent discharge
  - Other, please specify
  - No problems encountered
Q20. Do you believe route of placement for a cerclage (vaginal versus abdominal) has an effect on fertility?
  - Yes If so, please specify:
  - No
  - I don’t know
Q21. Do you believe route of placement for a cerclage (vaginal versus abdominal) has an effect on live obstetric outcomes?
  - Yes
  - No
  - I don’t know
Q22. Do you believe approach of trachelectomy (vaginal, abdominal, or laparoscopic) has an effect on survival?
  - Yes
  - No.
  - I don’t know
Q23. In your opinion, what is the main reason for preterm birth after a radical trachelectomy?
  - Cervical insufficiency
  - Infection
  - Other:
Q24. If you have further comments/questions regarding the questionnaire, please specify below.
Further three questions following feedback from respondents.
Q25. What do you believe is the optimal timing of cerclage placement after trachelectomy?
  - Immediately following trachelectomy
  - When there are signs of cervical insufficiency during pregnancy.
  - Other:
Q26. What is your preferred route of placement of a cerclage at time of trachelectomy?
  - Vaginally
  - Abdominally
Q27. What is your preferred route of placement of a cerclage for delayed placement (during pregnancy)?
  - Vaginally
  - Abdominally
